# Preoperative prediction of histopathological grading in patients with chondrosarcoma using MRI-based radiomics with semantic features

**DOI:** 10.1186/s12880-024-01330-4

**Published:** 2024-07-11

**Authors:** Xiaofen Li, Jingkun Zhang, Yinping Leng, Jiaqi Liu, Linlin Li, Tianyi Wan, Wentao Dong, Bing Fan, Lianggeng Gong

**Affiliations:** 1 1Department of Radiology, Jiangxi Provincial People’s Hospital, The First Affiliated Hospital of Nanchang Medical College, Nanchang, 330006 China; 2 2Department of Radiology, The Affiliated Hospital of Jiangxi University of Traditional Chinese Medicine, Nanchang, 330006 China; 3 Department of Medical Imaging Center, The Second Affiliated Hospital of Nanchang University, No.1 Minde Road, Donghu District, Nanchang, 330006 China

**Keywords:** Preoperative prediction, Chondrosarcoma grading, Nomogram, MRI radiomics, Semantic features

## Abstract

**Background:**

Distinguishing high-grade from low-grade chondrosarcoma is extremely vital not only for guiding the development of personalized surgical treatment but also for predicting the prognosis of patients. We aimed to establish and validate a magnetic resonance imaging (MRI)-based nomogram for predicting preoperative grading in patients with chondrosarcoma.

**Methods:**

Approximately 114 patients (60 and 54 cases with high-grade and low-grade chondrosarcoma, respectively) were recruited for this retrospective study. All patients were treated via surgery and histopathologically proven, and they were randomly divided into training (*n* = 80) and validation (*n* = 34) sets at a ratio of 7:3. Next, radiomics features were extracted from two sequences using the least absolute shrinkage and selection operator (LASSO) algorithms. The rad-scores were calculated and then subjected to logistic regression to develop a radiomics model. A nomogram combining independent predictive semantic features with radiomic by using multivariate logistic regression was established. The performance of each model was assessed by the receiver operating characteristic (ROC) curve analysis and the area under the curve, while clinical efficacy was evaluated via decision curve analysis (DCA).

**Results:**

Ultimately, six optimal radiomics signatures were extracted from T1-weighted imaging (T1WI) and T2-weighted imaging with fat suppression (T2WI-FS) sequences to develop the radiomics model. Tumour cartilage abundance, which emerged as an independent predictor, was significantly related to chondrosarcoma grading (*p* < 0.05). The AUC values of the radiomics model were 0.85 (95% CI, 0.76 to 0.95) in the training sets, and the corresponding AUC values in the validation sets were 0.82 (95% CI, 0.65 to 0.98), which were far superior to the clinical model AUC values of 0.68 (95% CI, 0.58 to 0.79) in the training sets and 0.72 (95% CI, 0.57 to 0.87) in the validation sets. The nomogram demonstrated good performance in the preoperative distinction of chondrosarcoma. The DCA analysis revealed that the nomogram model had a markedly higher clinical usefulness in predicting chondrosarcoma grading preoperatively than either the rad-score or clinical model alone.

**Conclusion:**

The nomogram based on MRI radiomics combined with optimal independent factors had better performance for the preoperative differentiation between low-grade and high-grade chondrosarcoma and has potential as a noninvasive preoperative tool for personalizing clinical plans.

**Supplementary Information:**

The online version contains supplementary material available at 10.1186/s12880-024-01330-4.

## Introduction

Chondrosarcoma is considered the most common primary bone neoplasm in adults, accounting for approximately 40% of all incidences of primary bone neoplasms. In children, its incidence is approximately 9.2%, second only to osteosarcoma among all primary malignant bone neoplasms, in which the pelvis and limb long bones are the most susceptible locations [1–5]. The new edition of the WHO bone tumour classification (2020) classifies conventional chondrosarcoma into three grades, namely, I, II, and III, based on the abundance and degree of atypia of chondrocytes, quantity of binuclear cells, nuclear division phases, and degree of mucus degeneration [6]. In particular, these guidelines classify grade I chondrosarcoma as a malignant tumour. This was formerly known as an intermediate type tumour.

At present, clinical treatment approaches for chondrosarcoma have mainly relied on histological grade and tumour location. Neoplasm resistance to chemotherapy and radiotherapy has contributed to its vascular deficiency and low proportion of dividing cells. To date, surgical resection remains the primary clinical treatment [7]. However, the surgical methods and prognosis of neoplasms primarily depend on their grades and biological behaviour [8–10]. The 2020 edition of the National Comprehensive Cancer Network (NCCN) guidelines recommend intralesional curettage as an alternative to extensive resection for chondrosarcoma patients with low-grade (grade I) disease, for achieving a positive prognosis. However, lesion scraping in combination with cryotherapy has been associated with a reduced recurrence rate for compartmental grade I chondrosarcoma. Some scholars have found that the 10-year survival rate of patients with grade I chondrosarcoma was reduced from 92–62%,while that for high-grade chondrosarcoma was only approximately 26% [11]. Chondrosarcoma is accompanied by a fairly high postoperative recurrence rate, and long-distance metastasis usually requires extensive and margin-negative resection or neoadjuvant therapy for treatment. In general, preoperative biopsy and radiological imaging are used to evaluate patients for surgical selection. However, the biopsy of efficacy is often limited by a small sample volume, which may cause under- or overdiagnosis of neoplasms [12].

MRI is considered an important examination procedure, owing to its high resolution on soft tissues and ability to reveal morphological features, such as periosteal reaction, cortical thickening, osmotic destruction, bone marrow oedema, deep scalloping, soft tissue mass and oedema, calcification shape, tumour cartilage abundance and entrapped fat within the tumour [13, 14]. These imaging characteristics,, which have been subsequently termed semantic features, have been shown to contribute to the diagnosis of chondrosarcoma. Numerous studies have shown that a higher grade chondrosarcoma was associated with low levels and blurred calcification, as well as obvious periosteal reaction and decreased tumour cartilage abundance [15]. Tumour cartilage abundance refers to the proportion of interstitial tissue surrounding tumour cells; on T2-weighted imaging (T2WI), areas of hyperintensity represent the cartilage matrix in the background of a heterogeneous tumour signal.

Conventional radiology inevitably has some difficulty in discriminating low- from high-grade chondrosarcoma [16]. Notably, radiomics allows the conversion of imaging data into spatial features with high-resolution and high-throughput quantitative information [17–19]. Previous studies have demonstrated that radiomics features based on CT/MR imaging effectively differentiate enchondroma from chondrosarcoma [20, 21]. To date, however, only a handful of studies have described the use of radiomics in combination with semantic features to correlate and discriminate different chondrosarcoma grades. The present study aimed to unravel the underlying association between radiomics and pathophysiology using a model to predict preoperative chondrosarcoma grading.

## Methods

### Ethics

This retrospective study was approved by the institutional review board, and informed consent was waived.

### Patient data

We retrospectively analysed 136 chondrosarcoma patients who were confirmed by surgical pathology at three tertiary bone tumour centres between January 2010 and May 2022. The inclusion criteria were as follows: (i) imaging including at least MRI scanning with T1-weighted imaging (T1WI) and T2-weighted imaging with fat suppression (T2WI-FS) sequences performed 1 month before clinical surgery; and (ii) definitive pathological diagnosis. Conversely, participants were excluded if (i) their image quality was poor or incomplete and thus could not be used to delineate the region of interest (ROI), making extracting imaging features difficult; (ii) they had secondary tumours or primary tumours in addition to with other tumours; (iii) they received other treatments before the MR scan, such as radiotherapy and chemotherapy; or (iv) they had tumours with pathological bone fracture. Ultimately, 114 chondrosarcoma patients were included in the study, 52 male and 62 female. The study group had an average age of 49.7 ± 17 (range 4 to 83) years. Grade I chondrosarcoma was categorized as a low-grade chondrosarcoma, while grade II and III chondrosarcomas were categorized as high-grade chondrosarcomas according to pathological grading. All chondrosarcoma patients were randomly distributed into training and validation sets at a ratio of 7:3.

### MRI examination and imaging evaluation

All patients included in the study underwent 3.0T MR scan (SIMENS MAGNETOM SKYRA 3.0T, GE DISCOVERY 3.0T), including at least T1WI and T2WI-FS sequences. The machine was set with the following scanning parameters [21]: [1] T1WI sequence: repetition time (TR): 619 ms, echo time (TE): 12 ms, matrix: 384 × 384, slice thickness: 5 mm, slice gap: 1 mm, flip angle: 160°, echo chain: 3 [2]. T2WI-FS sequence: TR: 3400 ms, TE: 85 ms, matrix: 384 × 384, slice thickness: 5 mm, slice gap: 1 mm, flip angle: 160°, and echo chain: 15. Semantic features were analysed by two musculoskeletal radiologists with at least 10 years of experience who were blinded to both the clinical information and pathology of the patient. The semantic features analysed included periosteal reactions, cortical thickening, intratumoral haemorrhage or necrosis, tumour cartilage abundance, bone marrow oedema, osmotic destruction, endosteal scalloping, soft tissue mass and oedema, maximum diameter, entrapped fat within the tumour, and calcification shape. MR imaging semantic features and clinical characteristics (sex and age) from patients in the training set were subjected to univariate logistic regression analysis, and those with statistically significant differences (*P* < 0.05) between low- and high-grade chondrosarcoma patients were subjected to multivariate logistic regression analysis to reveal independent factors closely correlated with chondrosarcoma grading.

### Tumour segmentation

The two musculoskeletal radiologists manually delineated each tumour lesion and then generated 3D-ROIs layer-by-layer using the freely open-source software ITK-SNAP (version 3.8.0, www.itksnap.org) on axial images as the preferred option, while considering coronal or sagittal sequences as the second option. Next, we applied the intraclass correlation coefficient (ICC) to evaluate the consistency in the ROI features delineated by the musculoskeletal physicians. Radiomics signatures with an ICC higher than 0.75 were considered eligible for further analysis. The delineated content included solid components and areas of calcification, haemorrhage and necrosis in the lesion.

### Image processing and feature extraction

We imported the 3D-ROI file and the corresponding original image into the Artificial Intelligence Kit Version 3.0.1 (Life Sciences, GE Healthcare, US) for feature extraction. Prior to feature extraction, we resampled and normalized the grey level as follows: the pixels were resampled to a 1*1*1 mm in-plane resolution, Then a Gaussian filter with the standard deviation of 0.5 was applied for signal smoothing, while the greyscale values were discretization from 0 to 255, after Laplacian of Gaussian (sigma = 2.0,3.0) filtering and wavelet decomposition (all combinations of high and low-pass filtering on the x and y axes).

Finally, we extracted a total of 1316 image quantitative imaging features, including 252 first order statistical features, 14 morphological features (Shape), 336 grey level cooccurrence matrix (GLCM) features, 196 grey level dependency matrix (GLDM) features, 224 grey run-length matrix (GLRLM) features, 224 grey level size zone matrix (GLSZM) features, and 70 neighbourhood grey tone difference matrix (NGTDM) features.

### Rad-score and nomogram construction

Next, we utilized the maximum correlation minimum redundancy (MRMR) algorithm to obviate the redundancy between features. Finally, the least absolute shrinkage and selection operator (LASSO) algorithm was used to reduce feature dimensionality and further select robust radiomics features. The rad-score of each patient was calculated using the following formula, and the results were subsequently used to construct the model: Rad_score = 0.0921870165791914*(Intercept)+-0.492678568393324*lbp_3D_k_glrlm_ShortRunHighGrayLevelEmphasis_T1+-0.56839426018634*log_sigma_2_0_mm_3D_firstorder_Skewness_T1 + 1.34754431549147*wavelet_HHL_firstorder_Skewness_T1+-0.288641675392167*wavelet_HHL_gldm_DependenceNonUniformityNormalized_T1+-0.522727858544381*wavelet_LHL_firstorder_Skewness_T2 + 0.961434335609768*wavelet_LHH_ngtdm_Contrast_T2。.

The clinical model was developed using the best independent semantic feature, while a nomogram was drawn integrating the optimal semantic feature with the rad-score by a multivariate logistic regression method. Model performance was assessed by the Hosmer‒Lemeshow test, analysing and comparing different models for preoperatively predicting chondrosarcoma grading. ROC curve analysis was performed, and the AUC was calculated to assess the discriminability of different models. The net benefits of the models were evaluated using decision curve analysis (DCA).

### Statistical analysis

All statistical analyses were performed using the statistical software SPSS 22.0 and packages implemented in R software version 4.1.0. Continuous variables are presented as the means ± standard deviations deviation (SD) or median (with lower and upper quartiles). Statistical comparisons between groups were performed using the Wilcoxon test, while the nomogram and calibration curves were constructed using the “rms” package. ROC curve analysis was used to determine the accuracy, sensitivity and specificity of the different models in predicting chondrosarcoma grading. The DeLong test was used to compare differences between the AUCs of the three models. For all statistical tests, *P* < 0.05 was considered to indicate statistical significance.

## Results

### Patient characteristics and semantic features

Distribution profiles of clinical semantic features in patients with different grades of chondrosarcoma are summarized in Table 1. The results from univariate and multivariate regression of the clinical and MRI semantic features of our patient lesion grade are outlined in Table 2.

**Table 1:** Clinical and semantic features in the training and validation sets

Clinical / semantic features	Training set (*n* = 80)		*p*-value	Validation set (*n* = 34)		*p*-value
	Low grade (*n* = 38)	High grade (*n* = 42)		Low grade (*n* = 16)	High grade (*n* = 18)	
**Gender**						
Male	14(36.8)	22 (52.4)		8(50.0)	8(44.4)	
Female	24(63.2)	20 (47.6)	0.242	8(50.0)	10(55.6)	1
Age (mean ± SD)	47.9 (16.5)	48.7 (17.7)	0.837	51.4 (16.5)	53.9 (17.4)	0.659
**Hemorrhage or necrosis**						
None	26 (68.4)	38 (90.5)		10 (62.5)	18 (100.0)	
Yes	12 (31.6)	4 (9.5)	0.029	6 (37.5)	0 (0.0)	0.016
Calcification shape	1.1 (0.9)	0.8 (0.5)	0.119	1.2 (0.9)	1.1 (0.4)	0.579
Maximum diameter (cm sd)	8.9 (5)	6.1 (3)	0.001	9 (4.1)	6.3 (3.4)	0.041
**Osmosis destruction**						
Yes	15 (39.5)	3 (7.1)		7 (43.8)	0 (0.0)	
None	23 (60.5)	39 (92.9)	0.001	9 (56.2)	18 (100.0)	0.006
Endosteal scalloping	0.8 (0.4)	0.7 (0.5)	0.617	0.9 (0.3)	0.7 (0.8)	0.317
Tumor cartilage abundance	2.1 (0.8)	2.6 (0.7)	0.003	2.1 (0.9)	2.8 (0.7)	0.012
**Periosteal reaction**						
None	25 (65.8)	39 (92.9)		6 (37.5)	18 (100.0)	
Yes	13 (34.2)	3 (7.1)	0.006	10 (62.5)	0 (0.0)	0.0003
**Cortical thickening**						
None	36 (94.7)	38 (90.5)		14 (87.5)	13 (72.2)	
Yes	2 (5.3)	4 (9.5)	0.766	2(12.5)	5 (27.8)	0.499
**Bone marrow edema**						
Yes	20 (52.6)	7 (16.7)		11 (68.8)	1 (5.6)	
None	18 (47.4)	35 (83.3)	0.0015	5 (31.2)	17 (94.4)	0.0004
**Soft tissue edema**						
Yes	23 (60.5)	9 (21.4)		13 (81.2)	4 (22.2)	0.0019
None	15 (39.5)	33 (78.6)	0.008	3 (18.8)	14 (77.8)	
**Soft tissue mass**						
Yes	22 (57.9)	14 (33.3)		7 (43.8)	2 (11.1)	
None	16 (42.1)	28 (66.7)	0.048	9 (56.2)	16 (88.9)	0.078
**Entrapped fat**						
None	34 (89.5)	33 (78.6)		2 (12.5)	2 (11.1)	
Yes	4 (10.5)	9 (21.4)	0.309	14 (87.5)	16 (88.9)	1
Rad-score	-0.35 (-0.70,0.17)	0.50(0.07,0.90)	*p* < 0.001	-0.19 (-0.60,0.15)	0.51 (0.11,0.73)	0.001

**Table 2:** Results from univariate and multivariate regression of clinical and MRI semantic features

Variable	Univariate regression		Multivariate regression	
	OR (95%CI)	*P*-value	OR (95%CI)	*P*-value
Gender	1.885[0.770–4.615]	0.165	NA	NA
Age	1.002[0.977–1.029]	0.835	NA	NA
Hemorrhage or necrosis	0.228[0.066–0.785]	0.019	0.34[0.09–1.34]	0.124
Calcification shape	0.604[0.318–1.147]	0.124	NA	NA
Maximum diameter	0.836[0.737–0.946]	0.005	NA	NA
Osmosis destruction	0.118[0.031–0.415]	0.001	NA	NA
Endosteal scalloping	0.778[0.294–2.059]	0.614	NA	NA
Tumor cartilage abundance	2.379[1.275–4.438]	0.006	1.95[1.01–3.74]	0.046
Periosteal reaction	0.148[0.038–0.572]	0.005	0.3[0.06–1.44]	0.132
Cortical thickening	1.895[0.327–10.986]	0.476	NA	NA
Bone marrow edema	0.180[0.064–0.505]	0.001	NA	NA
Soft tissue edema	0.178[0.067–0.475]	< 0.001	0.33[0.11,1.04]	0.058
Soft tissue mass	0.364[0.146–0.902]	0.029	NA	NA
Entrapped fat	2.319[0.650–8.267]	0.195	NA	NA

### Radiomics features and model construction

**Fig. 1:**
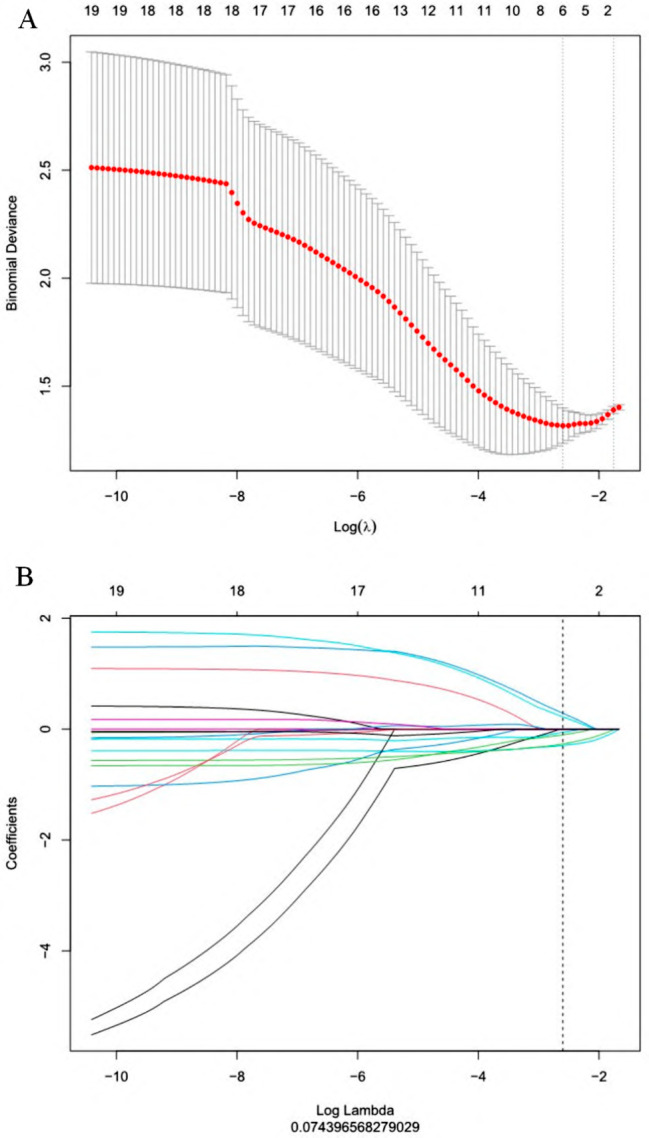
Results from the LASSO algorithm for radiomics feature selection. (**A**) Mean square error path using 10-fold cross validation; (**B**) LASSO coefficient profiles of the radiomics feature

**Fig. 2:**
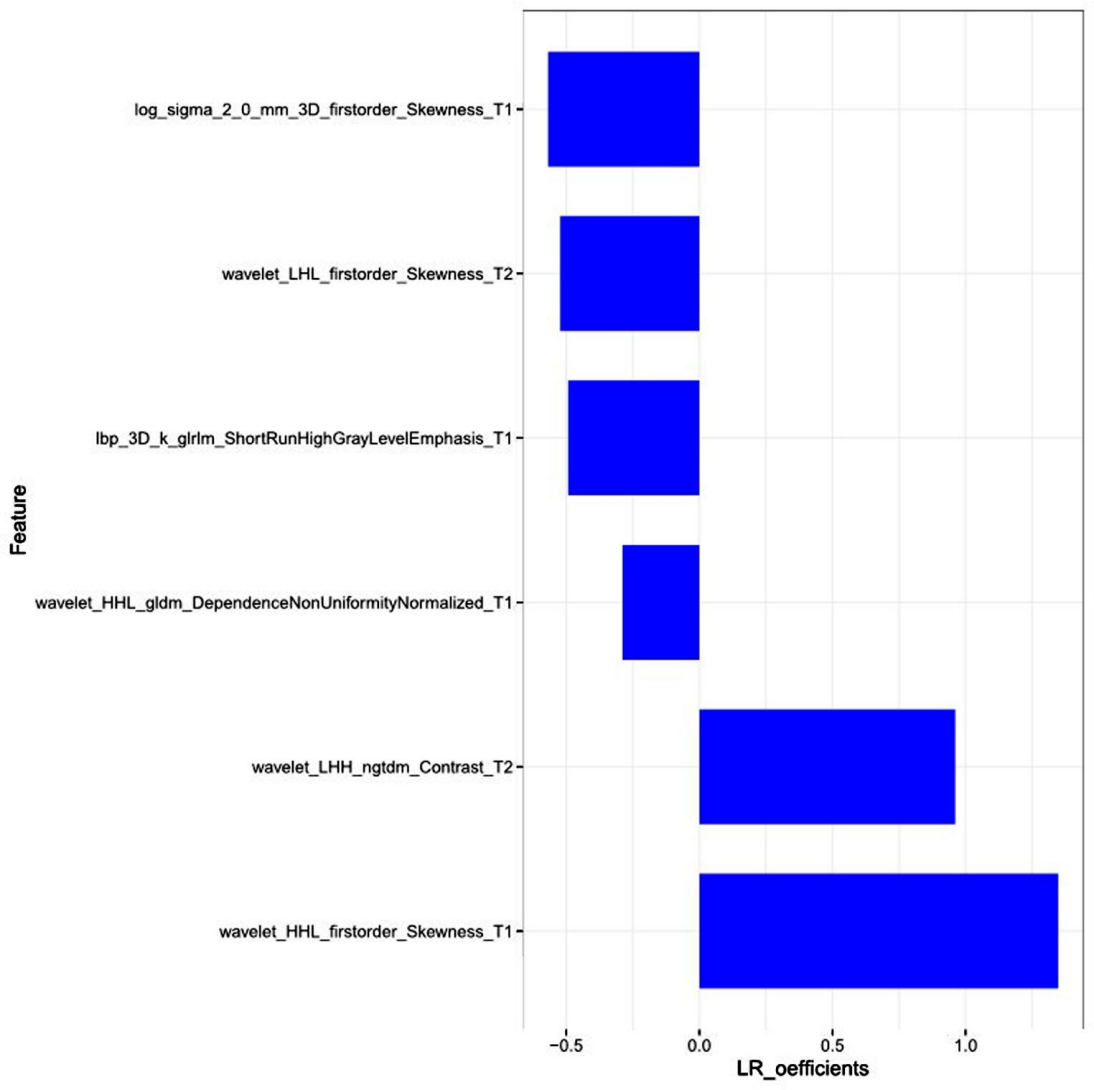
Radiomics feature selected by Logistics Regression

**Fig. 3:**
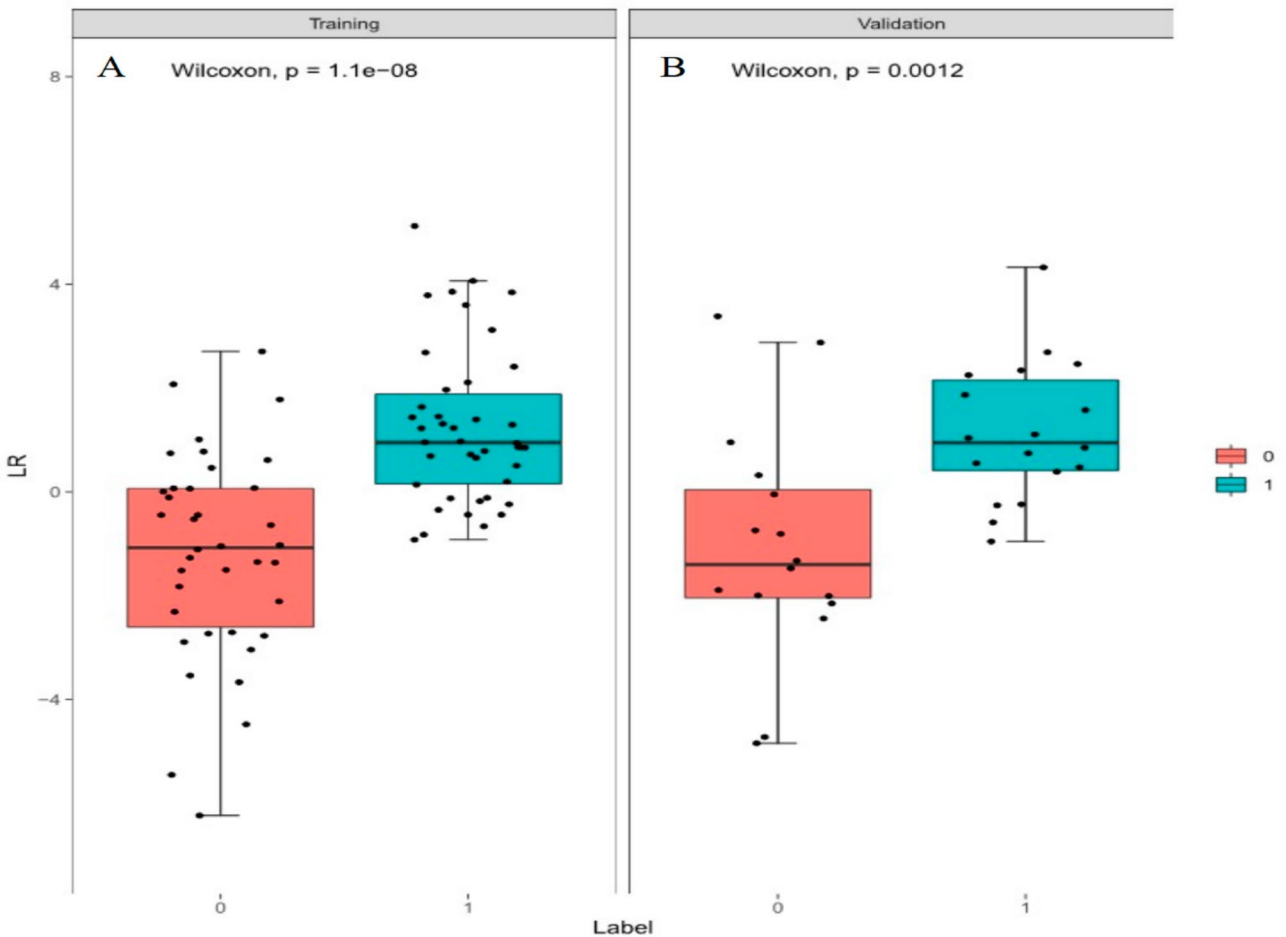
A scatterplot of Rad score in the training sets (**A**) and validation sets (**B**) (Label 0 and Label 1 correspond to low- and high-grade chondrosarcoma)

**Fig. 4:**
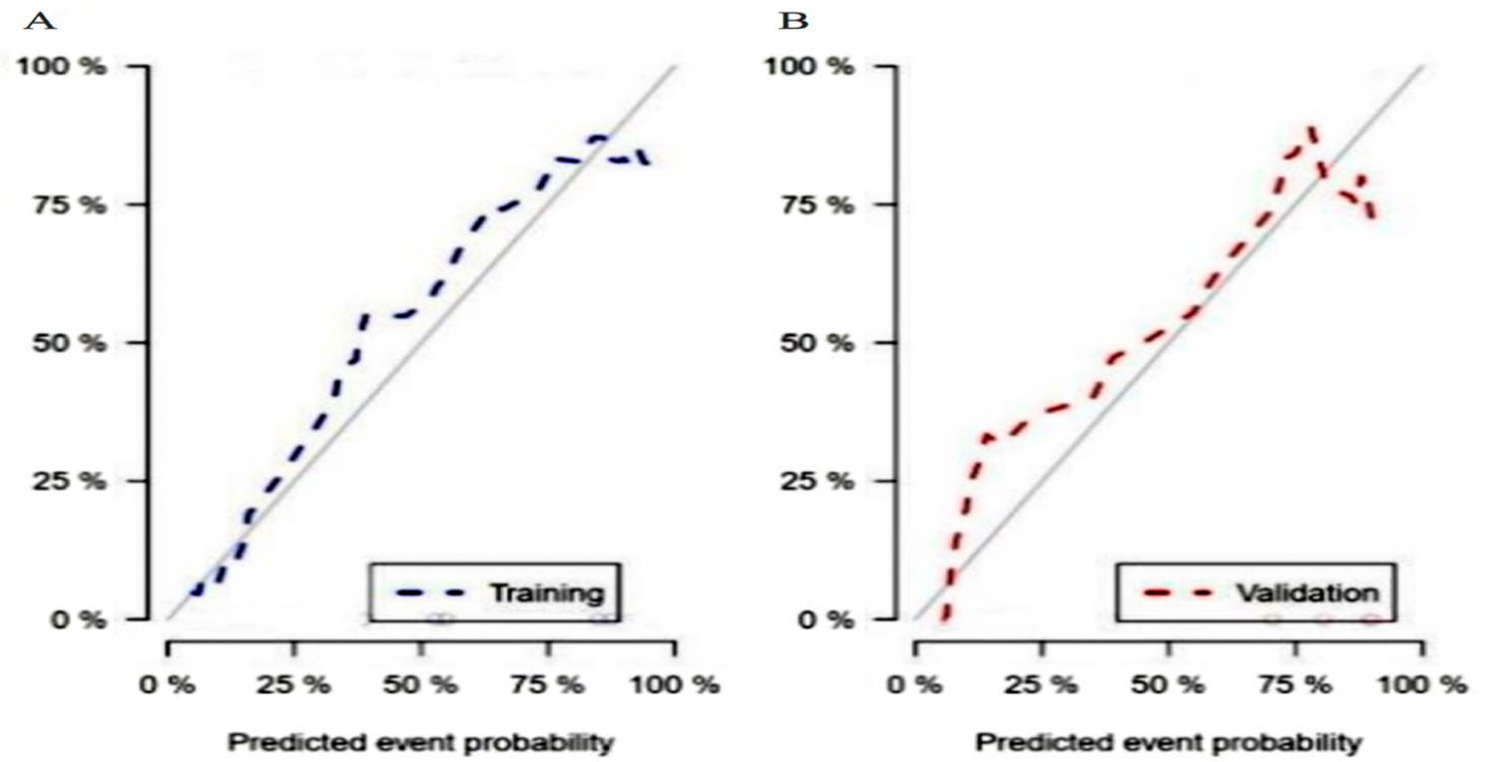
Calibration curves of the nomogram in the training sets (**A**) and validation sets (**B**)

A total of 1316 radiomics features were extracted from neoplasms in the training set. We applied the Spearman method to eliminate redundancy among these features. Finally, 6 optimal features were selected with high predictive value via LASSO regression (4 features from T1WI and 2 features from T2WI-FS sequences) (Fig. 1). We also obtained 3 first-order and 3 higher-order features (1 GLRLM, 1 GLDM, and 1 NGTDM) *(*Fig. 2). The radiomic model developed by the logistic classifier achieved a P value < 0.05 in both sets, indicating that it could significantly predict chondrosarcoma grading (Fig. 3). Next, we applied tumour cartilage abundance as an independent predictor to develop a clinical model and then employed the multivariable logistic regression algorithm to combine the rad-score and the best semantic signature for the preoperative grading of chondrosarcoma and intuitively establish a nomogram (Fig. 4). The results from the Hosmer‒Lemeshow test revealed no significant differences between the predicted outcome and the actual data, indicating that the radiomics nomogram had excellent fitting in both sets (*P* = 3.55, *P* = 5.23).

### Assessment of radiomic features

**Fig. 5:**
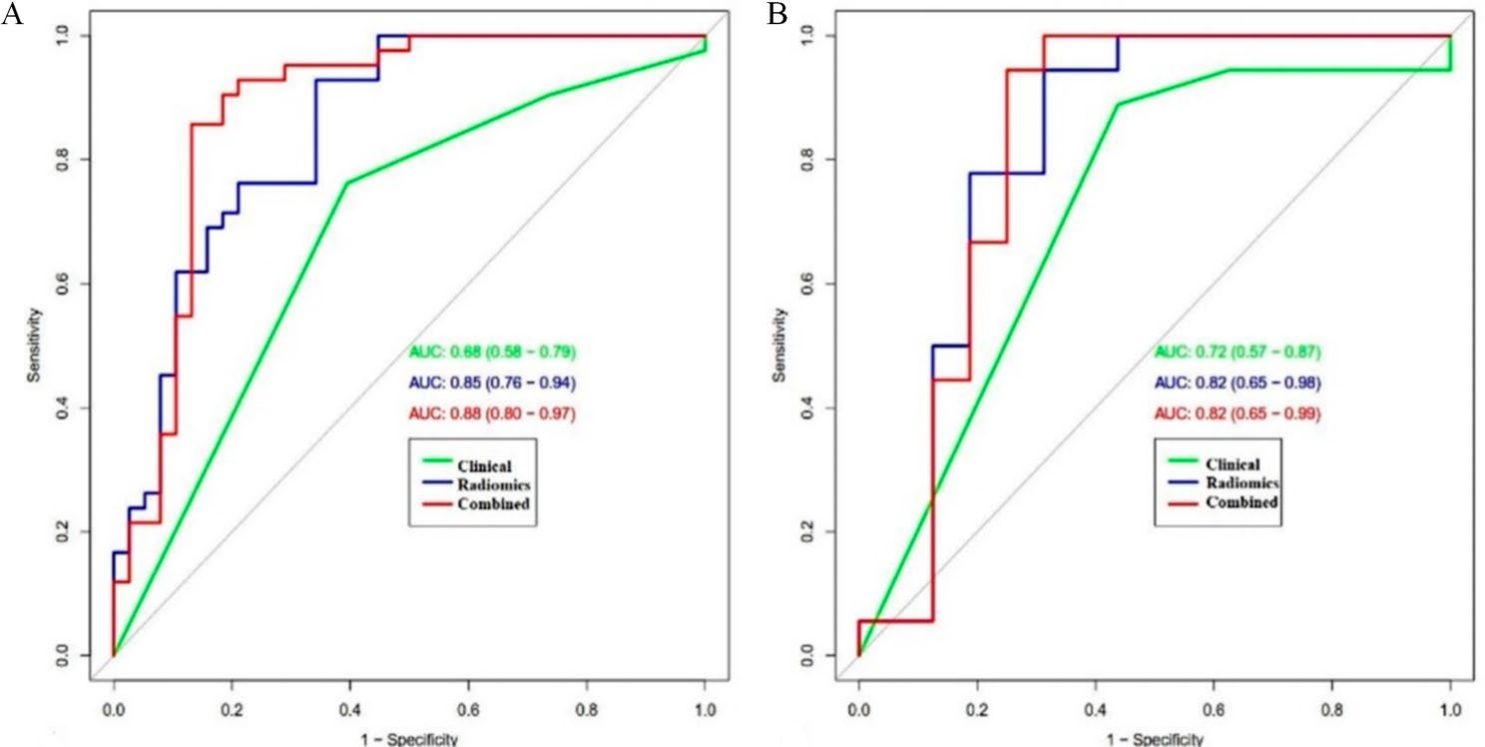
ROC curves of the Clinical, Radiomics, and Combined models in the training set (**A**) and validation set (**B**)

**Fig. 6:**
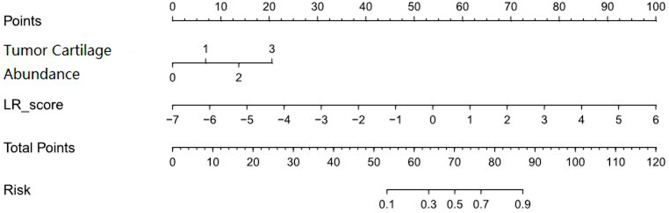
Radiomics nomogram with radiomics and semantic features. To draw an upward vertical line to the “Points” bar to calculate points. Based on the sum, draw a downward vertical line from the “Total Points” line to calculate the probability of classification of chondrosarcoma for each patient. For instance, grading in a 53-years-old woman with the Radscore value of 2.1,the tumor cartilage abundance value of 1.7, calculated from the formula, the corresponding value on the “Points” bar were 70 and 12.5, respectively. The probability of classification of chondrosarcoma was 84% by drawing a downward vertical line from the value of 82.5 on “Total Points” bar

**Fig. 7:**
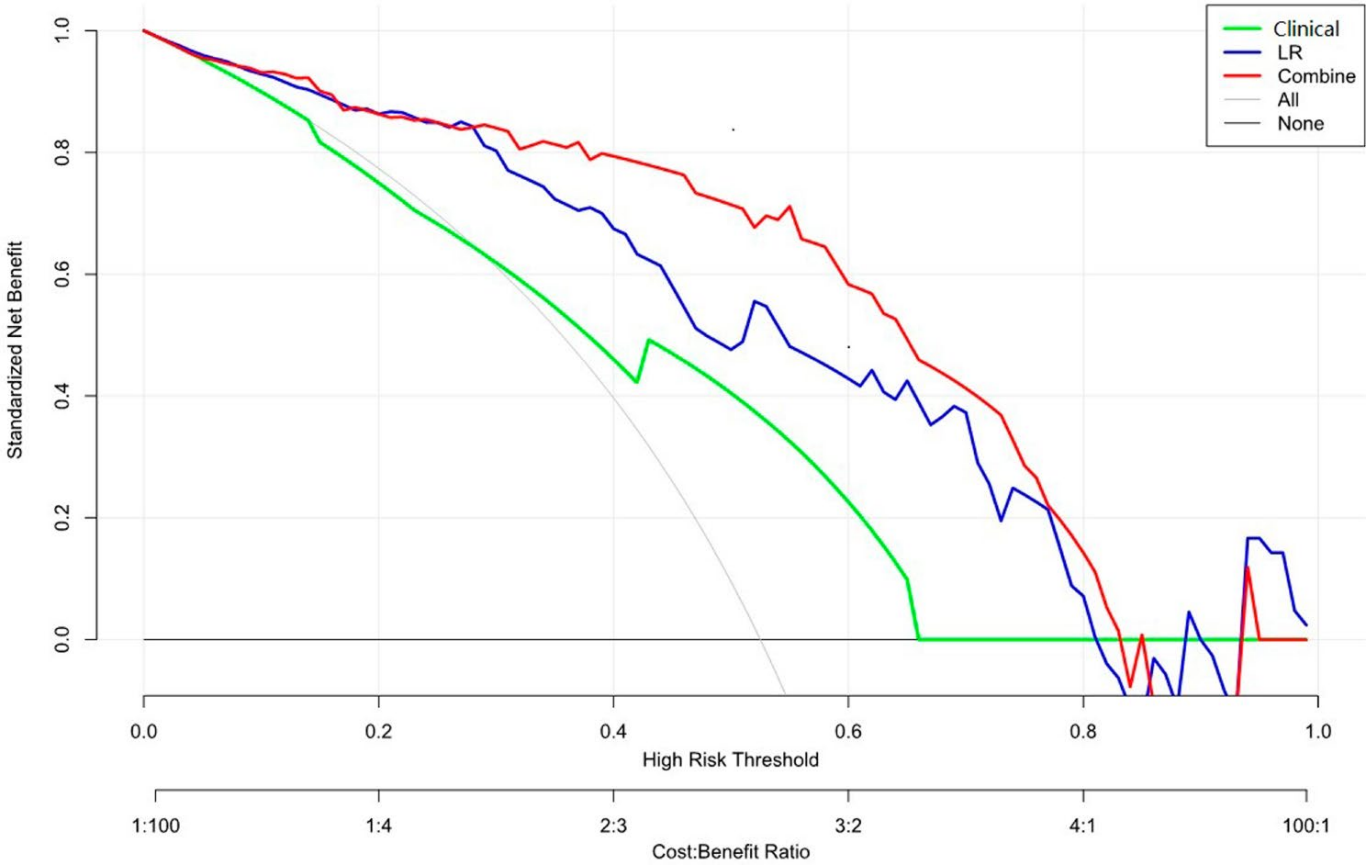
DCA of the radiomics nomogram. The y axis represents the net benefit, which was determined by calculating the difference between the expected benefit and the expected harm associated with each proposed model [net benefit = true-positive rate (TPR) – (false-positive rate (FPR)× weighting factor), where the weighting factor = threshold probability/ (1-threshold probability)]. The gray line represents the assumption that all tumors were high-grade, The black line represents the assumption that all tumors were low-grade

The ROC curves obtained for the three models are shown in Fig. 5. In summary, the combined model had the highest performance in the training set, as evidenced by an AUC value of 0.88 (95%CI: 0.80–0.97), followed by the radiomics model (AUC = 0.85, 95% CI: 0.76–0.94), whose AUC was significantly higher than that of the clinical model (AUC = 0.68, 95% CI: 0.58–0.79). Furthermore, the AUC values for the combined, clinical and radiomics models were 0.82 (95% CI: 0.65–0.99), 0.82 (95% CI: 0.65–0.98), and 0.72 (95% CI: 0.57–0.87), respectively, in the validation set. The results of the DeLong test revealed significant differences between the clinical model and the radiomic and combined models (Z=-2.561, *P* = 0.01045 and Z=-3.859, *P* = 0.0001, respectively) but not between the combined and radiomics models (*P* > 0.05). The accuracy, sensitivity, and positive predictive values of the three models are shown in Table 3. Notably, the nomogram model established herein had favourable discriminative consistency (Fig. 6*)*. In addition, the results of DCA showed that for threshold probabilities between 30% and 78%, the combined model had a markedly higher net benefit in the validation cohort than the clinical model, indicating that the nomogram had better clinical performance in predicting chondrosarcoma grading preoperatively (Fig. 7). In order to facilitate the repeatability and reproducibility of the study, we have provided a complete radiomics checklist [22] for study planning, manuscript writing, and evaluation in the Additional file 1: Table S1. According to the radiomics quality score (RQS) [23], the score for this study was 17 points. Detailed information about RQS was provided in the Additional file 1: Table S2.

**Table 3:** Predictive performance outcomes of the clinical, radiomics and combined models

	Model					
	Radiomics		Clinical		Combined	
	Training	Validation	Training	Validation	Training	Validation
Accuracy (95%CI)	0.8 (0.695–0.881)	0.794 (0.621–0.912)	0.687 (0.574–0.786)	0.471 (0.297–0.648)	0.862 (0.767–0.929)	0.823 (0.654–0.932)
Sensitivity (%)	92.8	88.8	76.2	73.0	85.7	88.8
Specificity (%)	65.7	68.7	60.5	1	86.8	75.0
Pos.Pred.Value	75.0	76.2	68.1	65.0	87.8	80.0
Neg.Pred.Value	89.2	84.6	69.7	47.1	84.6	85.7

## Discussion

In the present study, we constructed a combined radiomics nomogram that incorporated one optimal semantic feature with six robust radiomic features extracted from T1WI and T2WI-FS sequences for the efficient prediction of the grade of chondrosarcoma. The results showed that the nomogram has excellent performance in the validation set, indicating that the established radiomics model is considerably powerful and reliable.

Previous studies have shown that clinical treatment strategies for different grades of chondrosarcoma are dependent on accurate determination of its grade and the location of the neoplasm [24]. Grade I chondrosarcoma mainly requires intratumor curettage, owing to a local recurrence rate of 7.5–11%. Studies have shown that only 10% of all cases may develop into higher grade chondrosarcoma after recurrence, but the disease has a 10-year survival rate of approximately 92%, with a low metastasis rate [25–27]. On the other hand, extended resection or wide excision with negative margins has been recommended for the treatment of grade II/III chondrosarcoma and special types of chondrosarcoma due to the high metastasis rate and low 10-year survival rates [28, 29]. At present, there is a need to identify reliable and rigorous quantitative biomarkers to aid in discriminating low- from high-grade chondrosarcoma [30].

Recent radiomics studies have mostly focused on the differential diagnosis of enchondroma and chondrosarcoma, using different machine learning methods or only radiomics models [18, 20, 21, 31]. Other studies have focused on patient prognosis [32], but the prediction of chondrosarcoma grading has rarely been reported. Salvatore found that the logitboost classifier, based on CT imaging, was 81% (training set) and 75% (validation set) accurate in differentiating atypical cartilaginous tumours and higher-grade chondrosarcomas [18]. The authors also found an AUC and accuracy of 0.94 and 92% in the external validation set, respectively, with the Extra Trees classifier based on T1WI in one study comprising 158 patients with atypical cartilage tumours and chondrosarcoma grade II from two bone tumour centres, indicating that the model had excellent performance [20]. The results from a recent study confirmed that four features extracted from the T1WI sequence using the random forest algorithm had high diagnostic performance in distinguishing chondrosarcoma grade I/atypical cartilage tumours from high-grade chondrosarcoma, as evidenced by an AUC value of 0.78 in the validation cohort [31]. Moreover, Pan et al. developed clinical radiomics models that integrated tumour location with radiomics based on T1WI and T2WI-FS for discriminating enchondroma and chondrosarcoma [21].

Numerous studies have described the extraction of radiomics features from a single sequence, particularly T1WI. In the present study, we applied two sequences, namely, T1WI and T2WI-FS, and ignored dynamic contrast-enhanced scan or diffusion-weighted imaging, since some publications have revealed that these two techniques had no effect in differentiating the grading of cartilaginous tumours of different grades [33, 34]. In the present study, we selected a total of 6 radiomics signatures from T1WI + T2WI-FS sequences, consisting of 3 first orders (skewness) and 3 higher-order features (GLRLM, GLDM, and NGTDM). These radiomics features are typically utilized to describe the tumour matrix, as previously described [35], reflecting neoplasm heterogeneity. Skewness reflects the asymmetry of the histogram with respect to the mean, while GLRLM has been described as assessing heterogeneity, both in voxel intensities and spatial distribution, which was similar to the present study. Furthermore, the results of the present study showed that the logistic regression model based on the 6 radiomic features had high AUCs, indicating that it was efficient in differentiating low- from high-grade chondrosarcomas in long bones based on T1WI and T2WI-FS sequence features.

As we known, MR imaging has significant value in the differential diagnosis of bone tumors, MRI is the method of choice for local staging, while CT and PET-CT are employed for general staging [3]. Both MRI [5] and PET-CT based on standard uptake values [35] are accurate in discriminating chondrosarcomas grading. we visually classified the tumor signal of chondrosarcoma into low, medium, and high signals, which similar to adjacent normal muscle signals as low signals, similar to fluid signals as high signals, and intermediate signals as medium signals on T2WI sequence. Pathologically, the better the tumor differentiation, the closer it is to the originating tissue.the brighter the signal, the lower the tumor grade. However, these may cause subjective bias.

To further reveal its subtle correlation with the grading of chondrosarcoma, we attempted to introduce the concept of tumor cartilage abundance for quantitative analysis. In our result, analysis of the morphological MRI semantic features revealed that tumour cartilage abundance was the best independent risk factor for differentiating chondrosarcoma grading with *P* < 0.05. Next, we used this optimal semantic feature to construct a clinical model by multivariate logistic regression and found contrasting results from previous studies. For exam ple, Ban reported that cortical destruction and soft tissue mass were independent factors of high-grade chondrosarcoma [36], while Hassan showed that periosteal oedema was a morphologically independent factor for predicting chondrosarcoma grading after quantitative texture analysis of 116 patients [33]. We speculated that the discrepancy might be because their enrolled patients included fewer chondrosarcomas, which resulted in sample size bias, or the researchers might have ignored tumour cartilage abundance. In addition, the subjective difference between different protocols was based on qualitative observations, and researchers did not quantify heterogeneity among tumour characteristics. The clinical model had poor performance, as evidenced by AUC values of 0.72 and 0.68 in the training and validation sets, respectively. Fritz reported an AUC value of 0.84 in an MRI feature model incorporating active periostitis and maximum tumour extent, which was superior to our Model in differentiating chondrosarcoma grading [35].

Finally, although the performance of the combined model in the validation set was not as good as that of the radiomics model, the optimal clinical semantic feature also provided internal morphological information of the tumour to support the findings. On the other hand, we found that the combined model, which incorporated the optimal semantic signature, had better performance (AUC = 0.88, 95% CI: 0.80–0.97) than the single radiomics model alone in the training set, while the AUC in the validation set was equivalent to 0.82 in both models.

Our results showed that the radiomics model constructed based on T1WI and T2WI-FS sequences had considerable robustness, although the semantic features had little effect in improving model performance. These data indicated that the radiomics model is a powerful tool for predicting the pathological grading of chondrosarcoma preoperatively and thus could play a key role in guiding the development of personalized clinical treatment plans and improving the prognosis and survival rate of patients. In addition, we developed a nomogram model to guide clinicians when visually predicting chondrosarcoma grading. DCA showed that over the majority of thresholds, the models can achieve greater net benefits.

This study had some limitations. First, Although our images came from different devices in the same hospital and all data has been preprocessed, the differences of machines may result in subtle differences. In the future, enlarge sample size and more multicenter studies of external validation should be constructed to improve the robustness and generalization of the model; Second, the study employed a retrospective analysis approach, which is prone to selection bias. In the future, prospective randomized trials are needed to validate our findings. Third, this research involved a time-consuming manual delineation of the ROIs; thus, more advanced deep learning models are needed to validate our findings.Fourth, More multimodal parameters (such as: PET-CT, MRS) need to be included in our future study which can better reflect the heterogeneity of tumors.

In conclusion, a combination of clinical semantic features and radiomics has a strong predictive ability for the accurate preoperative grading of chondrosarcoma; thus, it can be used as a noninvasive, reliable and practical tool to guide the development of personalized clinical treatment plans.

### Electronic supplementary material

Below is the link to the electronic supplementary material.

Supplementary Material 1

## Data Availability

The datasets generated and/or analyzed in the current study are available upon request from the corresponding author.
