# Supplementary material for: Preoperative prediction of histopathological grading in patients with chondrosarcoma using MRI-based radiomics with semantic features
Source: BMC Med Imaging. 2024 Jul 11;24:171. doi: 10.1186/s12880-024-01330-4 (PMC11238384; doi:10.1186/s12880-024-01330-4)
Supplement: Supplementary file 1 — Supplementary Material 1 [file 12880_2024_1330_MOESM1_ESM.docx]

**Table S1.** Radiomics Research Evaluation Checklist (CLEAR Checklist).

| Section | No | Item | Yes | No | n/a | Page |
| --- | --- | --- | --- | --- | --- | --- |
| Title |  |  |  |  |  |  |
|  | 1 | Relevant title, specifying the radiomics methodology | √ |  |  | 1 |
| Abstract |  |  |  |  |  |  |
|  | 2 | Structured summary with relevant information | √ |  |  | 2 |
| Keywords |  |  |  |  |  |  |
|  | 3 | Relevant keywords for radiomics | √ |  |  | 3 |
| Introduction |  |  |  |  |  |  |
|  | 4 | Scientific or clinical background | √ |  |  | 3 |
|  | 5 | Rationale for using a radiomics approach | √ |  |  | 4 |
|  | 6 | Study objective(s) | √ |  |  | 5 |
| Method |  |  |  |  |  |  |
| Study Design |  |  |  |  |  |  |
|  | 7 | Adherence to guidelines or checklists (e.g., CLEAR checklist) | √ |  |  |  |
|  | 8 | Ethical details (e.g., approval, consent, data protection) | √ |  |  | 6 |
|  | 9 | Sample size calculation | √ |  |  | 6 |
|  | 10 | Study nature (e.g., retrospective, prospective) | √ |  |  | 6 |
|  | 11 | Eligibility criteria | √ |  |  | 6 |
|  | 12 | Flowchart for technical pipeline |  | √ |  |  |
| Data | 13 | Data source (e.g., private, public) | √ |  |  | 6 |
|  | 14 | Data overlap |  | √ |  |  |
|  | 15 | Data split methodology | √ |  |  | 6 |
|  | 16 | Imaging protocol (i.e., image acquisition and processing) | √ |  |  | 7 |
|  | 17 | Definition of non-radiomic predictor variables | √ |  |  | 7 |
|  | 18 | Definition of the reference standard (i.e., outcome variable) | √ |  |  | 7 |
| Segmentation | 19 | Segmentation strategy | √ |  |  | 8 |
|  | 20 | Details of operators performing segmentation | √ |  |  | 8 |
| Pre-processing | 21 | Image pre-processing details | √ |  |  | 8 |
|  | 22 | Resampling method and its parameters | √ |  |  | 8 |
|  | 23 | Discretization method and its parameters | √ |  |  | 8 |
|  | 24 | Image types (e.g.original, filtered, transformed) | √ |  |  | 8 |
| Feature extraction | 25 | Feature extraction method | √ |  |  | 8 |
|  | 26 | Feature classes | √ |  |  | 8 |
|  | 27 | Number of features | √ |  |  | 8 |
|  | 28 | Default configuration statement for remaining parameters | √ |  |  | 8 |
| Data preparation | 29 | Handling of missing data | √ |  |  | 8 |
|  | 30 | Details of class imbalance | √ |  |  | 8 |
|  | 31 | Details of segmentation reliability analysis | √ |  |  | 8 |
|  | 32 | Feature scaling details (e.g., normalization, standardization) | √ |  |  | 8 |
|  | 33 | Dimension reduction details | √ |  |  | 8 |
| Modeling | 34 | Algorithm details | √ |  |  | 9 |
|  | 35 | Training and tuning details | √ |  |  | 9 |
|  | 36 | Handling of confounders | √ |  |  | 9 |

**Table S1** (continued)

| Section | No | Item | Yes | No | n/a | Page |
| --- | --- | --- | --- | --- | --- | --- |
|  | 37 | Model selection strategy | √ |  |  | 9 |
| Evaluation | 38 | Testing technique (e.g., internal, external) | √ |  |  | 9 |
|  | 39 | Performance metrics and rationale for choosing | √ |  |  | 9 |
|  | 40 | Uncertainty evaluation and measure (e.g., confidence intervals | √ |  |  | 10 |
|  | 41 | Statistical performance comparison (e.g., DeLong’s test) | √ |  |  | 10 |
|  | 42 | Comparison with non-radiomics and combined methods | √ |  |  | 10 |
|  | 43 | Interpretability and explainability methods | √ |  |  | 10 |
| Results |  |  |  |  |  |  |
|  | 44 | Baseline demographic and clinical characteristics | √ |  |  | 10 |
|  | 45 | Flowchart for eligibility criteria | √ |  |  | 10 |
|  | 46 | Feature statistics (e.g., reproducibility, feature selection) | √ |  |  | 11 |
|  | 47 | Model performance evaluation | √ |  |  | 11-12 |
|  | 48 | Comparison with non-radiomic and combined approaches | √ |  |  | 12 |
| Discussion |  |  |  |  |  |  |
|  | 49 | Overview of important findings | √ |  |  | 12-13 |
|  | 50 | Previous works with differences from the current study | √ |  |  | 13-14 |
|  | 51 | Practical implications | √ |  |  | 14-15 |
|  | 52 | Strengths and limitations(e.g.bias and generalizability issues) | √ |  |  | 16 |
| Open Science |  |  |  |  |  |  |
| Data availability | 53 | Sharing images along with segmentation data [n/e] |  |  | √ |  |
|  | 54 | Sharing radiomics feature data |  |  | √ |  |
| Code availability | 55 | Sharing pre-processing scripts or settings |  |  | √ |  |
|  | 56 | Sharing source code for modeling |  |  | √ |  |
| Model availability | 57 | Sharing final model files |  |  | √ |  |
|  | 58 | Sharing a ready-to-use system [n/e] |  |  | √ |  |

**Table S2.** The radiomics quality score: RQS

| **Criteria** | | **Points** | **Score** |
| --- | --- | --- | --- |
| 1 | Image protocol quality - well-documented image protocols (for example, contrast, slice thickness, energy, etc.) and/or usage of public image protocols allow reproducibility/replicability | + 1 (if protocols are well-documented) + 1 (if public protocol is used) | **√** |
| 2 | Multiple segmentations - possible actions are: segmentation by different physicians/algorithms/software, perturbing segmentations by (random) noise, segmentation at different breathing cycles. Analyse feature robustness to segmentation variabilities | + 1 | **√** |
| 3 | Phantom study on all scanners - detect inter-scanner differences and vendor-dependent features. Analyse feature robustness to these sources of variability | + 1 | **×** |
| 4 | Imaging at multiple time points - collect images of individuals at additional time points. Analyse feature robustness to temporal variabilities (for example, organ movement, organ expansion/ shrinkage) | + 1 | **√** |
| 5 | Feature reduction or adjustment for multiple testing - decreases the risk of overfitting. Overfitting is inevitable if the number of features exceeds the number of samples. Consider feature robustness when selecting features | - 3 (if neither measure is implemented) + 3 (if either measure is implemented) | **√** |
| 6 | Multivariable analysis with non radiomics features (for example, EGFR mutation) - is expected to provide a more holistic model. Permits correlating/inferencing between radiomics and non radiomics features | + 1 | **√** |
| 7 | Detect and discuss biological correlates - demonstration of phenotypic differences (possibly associated with underlying gene–protein expression patterns) deepens understanding of radiomics and biology | + 1 | **×** |
| 8 | Cut-off analyses - determine risk groups by either the median, a previously published cut-off or report a continuous risk variable. Reduces the risk of reporting overly optimistic results | + 1 | **×** |
| 9 | Discrimination statistics - report discrimination statistics (for example, C‑statistic, ROC curve, AUC) and their statistical significance (for example, p‑values, confidence intervals). One can also apply resampling method (for example, bootstrapping, cross-validation) | + 1 (if a discrimination statistic and its statistical significance are reported) + 1 (if a resampling method technique is also applied) | **√** |
| 10 | Calibration statistics - report calibration statistics (for example, Calibration-in‑the-large/slope, calibration plots) and their statistical significance (for example, *P*‑values, confidence intervals). One can also apply resampling method (for example, bootstrapping, cross-validation) | + 1 (if a calibration statistic and its statistical significance are reported) + 1 (if a resampling method technique is also applied) | **√** |

**Table S2** (continued)

| 11 | **Prospective study** registered in a trial database - provides the highest level of evidence supporting the clinical validity and usefulness of the radiomics biomarker | + 7 (for prospective validation of a radiomics signature in an appropriate trial) | **×** |
| --- | --- | --- | --- |
| 12 | Validation - the validation is performed without retraining and without adaptation of the cut-off value, provides crucial information with regard to credible clinical performance | - 5 (if validation is missing) **+ 2 (if validation is based on a dataset from the same institute)** + 3 (if validation is based on a dataset from another institute) + 4 (if validation is based on two datasets from two distinct institutes) + 4 (if the study validates a previously published signature) + 5 (if validation is based on three or more datasets from distinct institutes)  *Datasets should be of comparable size and should have at least 10 events per model feature | **√** |
| 13 | Comparison to **‘gold standard**’ - assess the extent to which the model agrees with/is superior to the current ‘gold standard’ method (for example, TNM-staging for survival prediction). This comparison shows the added value of radiomics | + 2 | **√** |
| 14 | Potential clinical utility - report on the current and potential application of the model in a clinical setting (for example, **decision curve analysis**). | + 2 | **√** |
| 15 | Cost-effectiveness analysis - report on the cost-effectiveness of the clinical application (for example, QALYs generated) | + 1 | **-** |
| 16 | Open science and data - make code and data publicly available. Open science facilitates knowledge transfer and reproducibility of the study | + 1 (if scans are open source) + 1 (if region of interest segmentations are open source) + 1 (if code is open source) + 1 (if radiomics features are calculated on a set of representative ROIs and the calculated features and representative ROIs are open source) | **×** |
| **Total points (36 = 100%)** | | |  |
